# Preparation and Characterization of Beads of Sodium Alginate/Carboxymethyl Chitosan/Cellulose Nanofiber Containing Porous Starch Embedded with Gallic Acid: An In Vitro Simulation Delivery Study

**DOI:** 10.3390/foods11101394

**Published:** 2022-05-12

**Authors:** Wei Li, Wenxue Chen, Zhiyang Wang, Weijun Chen, Ming Zhang, Qiuping Zhong, Jianfei Pei, Haiming Chen

**Affiliations:** HNU-HSF Collaborative Innovation Laboratory, College of Food Sciences & Engineering, Hainan University, 58 People Road, Haikou 570228, China; 13778332220@163.com (W.L.); chwx@hainanu.edu.cn (W.C.); wzhiyang2022@163.com (Z.W.); chenwj@hainanu.edu.cn (W.C.); zhangming-1223@163.com (M.Z.); hainufood88@163.com (Q.Z.); peijianfei@hainanu.edu.cn (J.P.)

**Keywords:** porous starch, sodium alginate, cellulose nanofiber, carboxymethyl chitosan, gallic acid, small-intestine-targeted delivery

## Abstract

In this study, a system was designed that can encapsulate and deliver gallic acid (GA), which was composed of polysaccharide polymers based on sodium alginate (SA), carboxymethyl chitosan (CCT), and cellulose nanofibers (CN) and was assisted by porous starch. The compositions were characterized by rheology and zeta potentials, and the results showed that the materials used in this study could effectively guarantee the stability of the system. The morphology and chemical structure of the beads were characterized by SEM and FT-IR, the results indicated that the addition of CCT could effectively reduce the cracks and pores on the surface of the beads, which was beneficial to the encapsulation and delivery of GA. Moreover, the results of the swelling rate, release tests, and antioxidant tests also proved the effectiveness of the system. The pH response effect of SA/CN/CCT (SCC) beads and the protection of GA were superior, and the release rate of GA in simulated gastric fluid (SGF) was only 6.95%, while SA and SA/CN (SCN) beads reached 57.94% and 78.49%, respectively. In conclusion, the interpenetrating network polymers constructed by SA, CCT, and CN, which, combined with porous starch as a coating layer, can achieve the embedding and the delivery of GA.

## 1. Introduction

Due to changes in global diet, the incidence of obesity is increasing worldwide as is the incidence of glucose and lipid metabolism disorders, which are closely related to cardiovascular diseases, diabetes and fatty liver [1,2]. Gallic acid (3,4,5-trihydroxybenzoic acid, GA), a common dietary polyphenol, is widely found in foods such as tea and mango [3]. GA can ameliorate diet-induced glucose and lipid metabolism disorders through the regulation of energy metabolism and adipocyte differentiation and by promoting glucose absorption and utilization while increasing insulin sensitivity [4]. GA can also regulate the level of blood glucose and blood lipids by inhibiting the intestinal digestion and absorption of fat, reducing lipid synthesis and accumulation, and regulating gluconeogenesis and glycolysis [5]. Meanwhile, GA promotes mitochondrial energy metabolism and prevents DNA damage caused by a high-fat diet [6]. GA have many physiological functions; however, GA performs poorly in terms of bioavailability and stability and is easily destroyed; moreover, the application of GA is limited by its tendency to auto-oxidize and form dimer, oligomer, and polymers in aqueous solution [7]. In addition, GA is sensitive to the human digestive system environment, and the small intestine is difficult to efficiently transport GA, while the human diet environment is more complex; before reaching the absorption zone of GA, GA is very likely to be complexed into various degrees of polymerization by the upper digestive system, stomach, etc., or directly degraded to cause a great deal of loss [7]. In terms of the bioavailability of phenols, monomers have a higher absorption rate than polymers. Porous starch (PS) has abundant pores from the surface to the interior of the granule, which means it has a strong capacity for adsorbing GA monomers. The adsorption by PS may reduce the formation of GA polymers; therefore, the bioavailability of GA may increase as GA maintains the monomer’s condition [8]. However, PS would be almost completely hydrolyzed in the gastrointestinal (GI) tract, so it is difficult for the carried cargos to reach the target zone [9].

Sodium alginate is an anionic polysaccharide found in brown algae. Its carboxyl groups can cross-link with divalent cations (such as calcium ions) and form insoluble calcium alginate, which has been widely used in drug-delivery systems [10]. Cellulose nanofiber (CN) is a nano-level polymer material prepared from natural cellulose, which is usually defined as ultra-fine fiber with diameter < 100 nm and which has excellent mechanical properties and biodegradability [11]. The polyhydroxyl structure of CN can enhance the gel strength of hydrocolloids, such as protein gel by binding forces [12]. Therefore, the nanocellulose may strengthen the mechanical structure of sodium alginate gels, prevent the gel from rupture due to vigorous peristalsis of the GI tract, and help achieve sustainable release of the contents [13]. Surface positively charged chitosan can form gels with multivalent anions, such as alginate, by ionic cross-linking. Carboxymethyl chitosan (CCT) is a chitosan derivative with carboxymethyl substituents on both the amino and primary hydroxyl groups of the chitosan; as an amphoteric electrolyte, CCT can switch the surface charge between positive and negative when the environmental pH changes, so it can cross-link with non-toxic polyvalent anions such as sodium alginate to form gels, and it has been proven that CCT is highly water-soluble and pH-sensitive [14,15]. SA, CN and CCT can traverse the upper GI tract; then, they are decomposed by intestinal digestive enzymes secreted by the abundant bacteria in the GI tract. As the decomposition proceeds, the cargos are continuously released from the PS particles to achieve local target delivery. Polysaccharide-based microcapsules have robust capsule walls and rapid and sensitive response behavior, suggesting their great potential for targeted slow-release delivery [16]. Ca^2+^-SA based gels prepared by external gelation have been widely used in the food and pharmaceutical industries as a novel delivery vehicle for bioactive substances; nevertheless, hitherto, the effect of sodium alginate-nanocellulose-chitosan gels on the tolerance of gallic acid against auto-oxidation and self-aggregation in GI fluid has been rarely investigated [17].

This study revolves around the construction of a natural complex polysaccharide delivery system. Through the adsorption of GA by PS, the porous starch–GA assemblies are secondarily encapsulated to achieve targeted delivery and slow release of GA in the human intestine, using the pH sensitivity and mechanical properties of natural complex polysaccharide packages for protection purposes.

## 2. Materials and Methods

### 2.1. Materials

Porous starch (BET Surface Area: 17.3639 m^2^/g, BJH Adsorption cumulative volume of pores between 1.7000 nm and 300.0000 nm width: 0.021031 cm^3^/g) were purchased from Kangze Biotechnology Co., Ltd. (Xi’an, China). GA, SA (medium viscosity), CCT (BR, CAS: 83512-85-0), pepsin (1:15,000), and pancreatin from porcine pancreas (USP, CAS: 8049-47-6) were purchased from Aladdin Inc. (Shanghai, China). CN was purchased from Qihong Technology Co., Ltd. (Guilin, China).

### 2.2. Loading PS with GA

The loading process was performed according to a previously published protocol [18]. Dissolve the GA in distilled water and sonicate for 10 min to obtain a clear solution (10 mg/mL). Afterwards, PS powder was added to the GA solution (1.5:1, g/mL), and encapsulation was conducted at room temperature under magnetic shaking (85-1 Aohua Instrument, Changzhou, China). After shaking for 12 h, the slurry was centrifuged at 4000× *g* for 15 min, and the absorbance of the supernatant was measured at 760 nm with a UV–Vis spectrophotometer (TU 1810 SPC). The amount of GA was determined by the Folin–Ciocalteu assay [7] using a standard curve. The sediments were dried at 40 °C for 12 h and then stored in a desiccator. The encapsulation efficiency (EE) and loading capacity (LC) were calculated by the following Equations (1) and (2):

(1) $$\mathrm{EE}\;(\%)=\frac{M_{0}-M_{s}}{M_{0}}\times 100\%$$

(2) $$\mathrm{LC}\;(\%)=\frac{M_{G}}{M_{T}}\times 100\%$$

where M_0_ is the total weight of GA in the slurry, M_s_ refers to the weight in the supernatant, M_G_ is the weight of the PS/GA, and M_T_ is the total weight of the PS/GA.

### 2.3. Preparation of Beads

The beads were prepared according to the method of our group [18]. SA, CCT, and CN powders were weighed accurately and poured into distilled water with high-speed shearing. The PS/GA (PG) powders were dissolved in water and physically mixed with polysaccharide interpenetrating network polymers (PIPNs). Then, the mixed solution was degassed under vacuum, and the solution was further extruded through a syringe (5 mL) into the CaCl_2_ solution (2%, *w*/*v*). The freshly formed beads were immersed in the acidic CaCl_2_ solution for 30 min to promote complete gelatinization, and then, all beads were washed with distilled water twice. The GA content in the CaCl_2_ solution was determined to calculate the loss of GA [7].

Based on the single-factor experiments, four material variables were screened out (the concentration of CaCl_2_, SA, CN, and CCT). According to the Box–Behnken design, twenty-nine experimental runs were performed. The Design Expert 11.0 software (Stat-Ease, Inc., Minneapolis, MN, USA) was used to establish the mathematical progress. The optimum composition of the wall materials was determined by regression equation analysis.

### 2.4. Zeta-Potential

The zeta-potential of the GA, PS, PG, pure hydrocolloid solutions, and PIPNs/GA solutions were measured on a zeta potential analyzer (Zetasizer Nano S90, Malvern Instruments, Malvern, UK) according to the method of Zhou et al. [19]. The solutions were diluted 100 times with ultra-pure water before determination. All the measurements were performed in triplicate at 25 °C.

### 2.5. Rheology

The apparent viscosity of different samples was determined on a Thermo HAAKE Rotation Rheometer (HAAKE MARS 40, Karlsruhe, Germany) equipped with 35 mm parallel steel plates, and the gap between plates was 2.00 mm. SA solution (2.0 *w*/*v*%), SCN (SA/CN) solution (2 *w*/*v*% SA, 0.5 *w*/*v*% CN), SCT (SA/CCT) solution (2 *w*/*v*% SA, 0.5 *w*/*v*% CCT), and SCC (SA/CN/CCT) solution (2 *w*/*v*% SA, 0.5 *w*/*v*% CN, 0.5 *w*/*v*% CCT) were prepared, and the relationship between the apparent viscosity and shear rate (0.1~100 s^−1^) was evaluated.

### 2.6. Characterization of PG and Beads

#### 2.6.1. Texture Analysis

The texture profiles of the fresh beads were analyzed on a TA-XT plus texture analyzer (TA.XT.Plus texture analyzer, Stable Micro Systems, Godalming, UK) at 25 °C according to the method of Guo et al. [20]. Beads were compressed twice (1 mm/s) to 50% of their original heights by a load cell and a cylinder probe (P/36R, Stable Micro Systems).

#### 2.6.2. Scanning Electron Microscopy (SEM)

The freeze-dried beads were glued to the plate with double-sided adhesive and then sprayed with gold. The morphological characteristics of the samples were observed and photographed (Supra 55, Zeiss, Oberkochen, Germany) with an accelerating voltage of 10 kV [21].

#### 2.6.3. Fourier-Transform Infrared Spectroscopy (FT-IR) Analysis

To characterize the intermolecular interaction between SA, CCT, and CN as well as the hydrogen bond interaction between PS and GA, spectrograms were acquired using a FT-IR (TENSOR 27, Bruker Optics, Ettlingen, Germany) equipped with a deuterated triglycine sulfate detector. The dried powders of samples were compressed with KBr to form a disc shape that was scanned from 4000 to 400 cm^−1^ at a resolution of 4 cm^−1^. The absorption spectrum was obtained after denoising and baseline correction.

#### 2.6.4. Thermogravimetric Analysis (TGA)

The thermal stability of the beads and the PG powders were analyzed on a simultaneous thermal analyzer (Netzsch STA 449C, Aldridge, UK). Samples (10 mg) were placed on an aluminum pan (sealed immediately) and heated from 30 °C to 600 °C at a rate of 20 °C/min.

### 2.7. Swelling Rate

The pH sensitivity of beads was evaluated by measuring the swelling behavior according to the method of Sun et al. [22]. Dried SA, SCN, SCT, and SCC beads were precisely weighed (10 mg) and swollen at 37 °C under pH 1.2, 6.8, and 7.4 solutions, which were composed of HCl, KCl, Na_2_HPO_4_, and NaH_2_PO_4_, for 8 h in total. The swollen beads were weighed immediately after removing the liquid adhered. The swelling rate of the beads was calculated according to Equation (3):

(3) $$\mathrm{Swelling}\;\mathrm{rate}\;(\%)=\frac{W_{t}-W_{o}}{W_{o}}\times 100\%$$

where W_t_ is the weight of swollen beads at time t, and W_o_ is the initial weight of the beads.

### 2.8. Antioxidant Activity of PG and Beads

The antioxidant activity of the samples was estimated by their ability to scavenge DPPH radical (2,2-diphenyl-1-picrylhydrazyl), as previously described by Sun et al. [22]. Beads (0.05 g) were suspended in Milli-Q water (15 mL) for 4 h and then centrifuged at 4000× *g* for 10 min. The supernatants (2.0 mL) were added to DPPH solution (2.0 mL, 2mM). The absorbances of the sample DPPH-ethanol solution, DPPH-ethanol solution, and the sample ethanol solution were read as A_0_ and A_1_.

The results were expressed in percentage of inhibition (PI) of the DPPH radical (Equation (4)):

(4) $$\mathrm{PI}\;\left(\%\right)=\frac{A_{0}-\left(A_{S}-A_{1}\right)}{A_{0}}\times 100\%$$

### 2.9. In Vitro Release Study

The in vitro simulated digestion was carried out according to the method of Sun et al. [22]. The dried beads (100 mg) were immersed in different simulate digestive fluids (simulated gastric fluid (SGF)/simulated intestinal fluid (SIF)/simulated colonic fluid (SCF)) and Milli-Q water under gentle stirring (100 rpm) at 37 °C. Aliquots (1.0 mL) were taken at desired intervals, and an equivalent volume of fresh medium was supplemented. Then, the concentrations of GA were detected by the Folin–Ciocalteu assay [7].

The cumulative percentage of GA release was estimated under simulated gastrointestinal conditions by first incubating in a solution at pH (1.2) for 2 h at 37 °C under constant shaking (100 rpm), then in a solution at pH (6.8) for an additional 2 h, and finally in a solution at pH (7.4). The above steps were repeated on other beads.

Under simulated GI tract conditions, the cumulative GA release percentage was estimated by incubation at 37 °C under constant shaking (100 rpm) in a solution (pH 1.2) for 2 h firstly, then a solution (pH 6.8) for an additional 2 h, and lastly a solution (pH 7.4) for 2 h as well. The above procedures were repeated on the other beads.

### 2.10. Statistical Analysis

All the assays were replicated three times. Analyses of variance were evaluated by Duncan’s multiple-range test (*p* < 0.05) using SPSS 24.0 (SPSS Inc., Chicago, IL, USA), and the data are reported as mean values ± standard deviations. Origin Pro 9.0 (Origin Lab, Northampton, MA, USA) was used for data processing and chart creation.

## 3. Results and Discussions

### 3.1. Optimization of Encapsulation Efficiency

#### 3.1.1. Optimization of Loading PS with GA

An effective delivery system requires significant encapsulation efficiency and loading capacity. Hence, it is important to assess the encapsulation efficiency and loading capacity of a novel delivery system [22]. The weight ratio of PS to the GA as well as the encapsulation time and temperature were optimized to obtain an acceptable loading capacity (LC). The LC increased as the ratio of GA to PS increased from 0.2 to 0.66, reaching a maximum of 320.05 mg/g at room temperature for 12 h. A homothetic result was previously reported by our group [18], whose encapsulation efficiency of PS to doxorubicin peaked at 95.27% when the ratio of PS to doxorubicin was 0.75. Similar trends were previously reported for the adsorption of porous corn starch to methyl blue [23] and the adsorption of procyanidins to chitosan-modified porous rice starch [24].

#### 3.1.2. Optimization of Wall Material Composition

Based on the results of single-factor experiments, twenty-nine experiments were designed, and Box–Behnken design was performed to optimize the wall material compositions, and coded values and corresponding actual values of the optimization parameters used in response surface analysis are shown in Table 1.

Regression analysis was used to evaluate the experimental factor coding and results, and a significance test was conducted (Table 1). The encapsulation efficiency could be explained by quadratic regression as follows (Equation (5)):

(5) $$\begin{array}{c} Y\;\left(\%\right)=55.04+3.21*A-3.73*B-6.12*C+3.94*D-4.40*\mathrm{AB}-1.63*\mathrm{AC}-5.06*\mathrm{AD}-0.8375*\mathrm{BC}+1.06*\mathrm{BD}\\ +1.06*\mathrm{CD}-8.11*A^{2}-4.84*B^{2}-4.17*C^{2}-0.2177D^{2}-3.73*A^{2}B+5.23*A^{2}C-4.70*A^{2}D\\ -0.4325*{\mathrm{AB}}^{2}-0.4800*{\mathrm{AC}}^{2}+4.85*B^{2}C-5.00*B^{2}D+2.39*{\mathrm{BC}}^{2} \end{array}$$

where Y is the encapsulation efficiency for the beads, and A, B, C, and D are the coded variables.

The results of analysis of variance (ANOVA) for encapsulation efficiency of beads are summarized in Table 2. The F-value and *p*-value of the model were 9.05 and 0.0057, respectively, suggesting the significance of the model. The lack-of-fit was non-significant (*p* > 0.05), indicating that the encapsulation efficiency of wall materials can be accurately predicted using the quadratic model. C, AD, A^2^, B^2^, and C^2^ were found to be extremely significant terms, and B, D, AB, A^2^C, A^2^D, B^2^C, and B^2^D were significant model terms. The lack-of-fit was not significant relative to the pure error, as the corresponding F-value was 9.05. In addition, the coefficient of determination (R^2^) was 0.9708, and the low values of the coefficient of variation (5.50%) and adequate precision (11.3654) indicated a good model fit [25].

**Figure 1:**
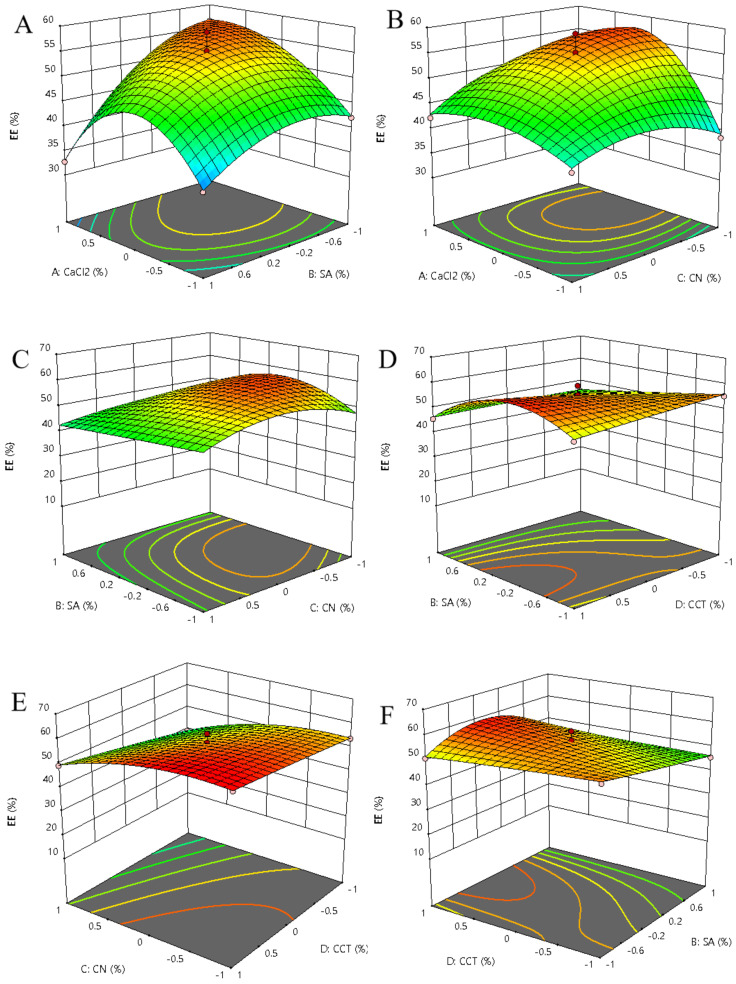
Response surface of the effects among various factors on the encapsulation efficiency of beads. (**A**) Interaction of CaCl_2_ and SA; (**B**) Interaction of CaCl_2_ and CN; (**C**) Interaction of SA and CN; (**D**) Interaction of SA and CCT; (**E**) Interaction of CN and CCT; (**F**) Interaction CCT and SA. SA: sodium alginate, CN: cellulose nanofibers, CCT: carboxymethyl chitosan, EE: encapsulation efficiency.

The three-dimensional response surfaces were built to explain the interactions from the four variables and determine the optimal combination. A clear interaction among CaCl_2_, SA, CN, and CCT was observed (Figure 1). The optimal concentration calculated from the regression equation (Equation 5) for the maximal predicted encapsulation efficiency (54.41%) was acquired: 2.37% CaCl_2_ (*w*/*w*), 2.19% SA (*w*/*w*), 0.43% CN (*w*/*w*), and 1.17% CCT (*w*/*w*). After validation, the encapsulation efficiency was found to be 56.57%, showing 96.19% agreement. Therefore, the optimal beads preparation scheme optimized by this model is reliable.

### 3.2. Characterization of PG, PIPNs and Beads

#### 3.2.1. Zeta-Potential

**Figure 2:**
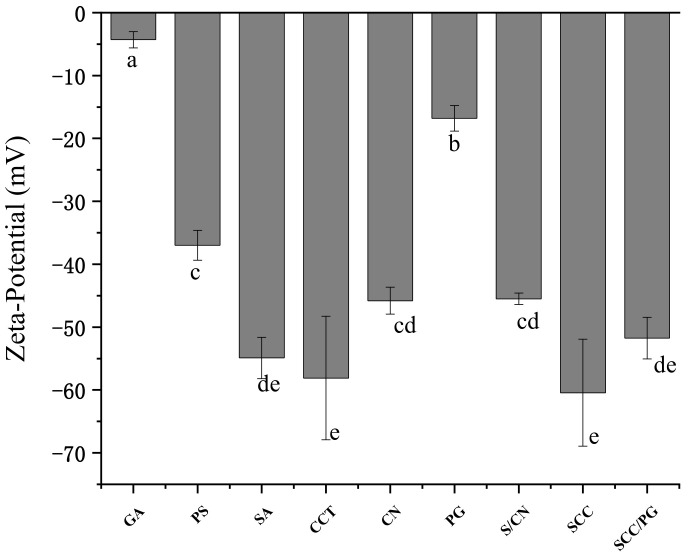
Zeta-potential of GA, PS, PG, pure hydrocolloid solutions, and PIPNs/GA solutions. Different lowercase letters indicate significant differences. GA: gallic acid, PS: porous starch, SA: sodium alginate, CCT: carboxymethyl chitosan, CN: cellulose nanofibers, PG: PS/GA, S/CN: SA/CN, PIPNs: polysaccharide interpenetrating network polymers, SCC: SA/CN/CCT.

As one of the important indications of charged polymers, zeta-potential reflects the interpenetration of the biopolymers, and the charged nature is the most significant [26,27]. The zeta-potentials of GA, PS, PG, SA, CN, CCT, and other polysaccharides are shown in Figure 2. When dissolved in water, GA was negatively charged and had a zeta-potential of −4.3 mV. After encapsulation with PS, GA was stabilized (a lower zeta-potential of PG). Though the zeta-potential of PG was higher than that of PS, the stability of PG still needs further improvement. The zeta-potential of SA, CN, and CCT was −54.9, −45.8, and −58.1 mV, respectively, and that of the physical mixture of SA and CN (SCN) was −45.5 mV, slightly higher than any single polysaccharide solution. After the addition of CCT and physical interpenetrating process, the zeta-potential of SCN decreased to −60.43 mV, revealing that the SCC system was extremely stable. The zeta-potential of SCC/PG system was increased slightly to −51.73 mV. The behavior of individual biopolymers is influenced by the presence of other biopolymers in solution once they are mixed and interpenetrated, as mutual masking of surface charges might occur among the multiple polymer solutions [28]. The results of zeta-potential analyses showed that the SCC could effectively provide protection for GA and ensure the stability of the system. Meanwhile, the coating layer provided by SCC and PS could guarantee the bioavailability of GA to some extent when GA suffers from extreme pH and peristalsis of gastrointestinal environment.

#### 3.2.2. Rheology

**Figure 3:**
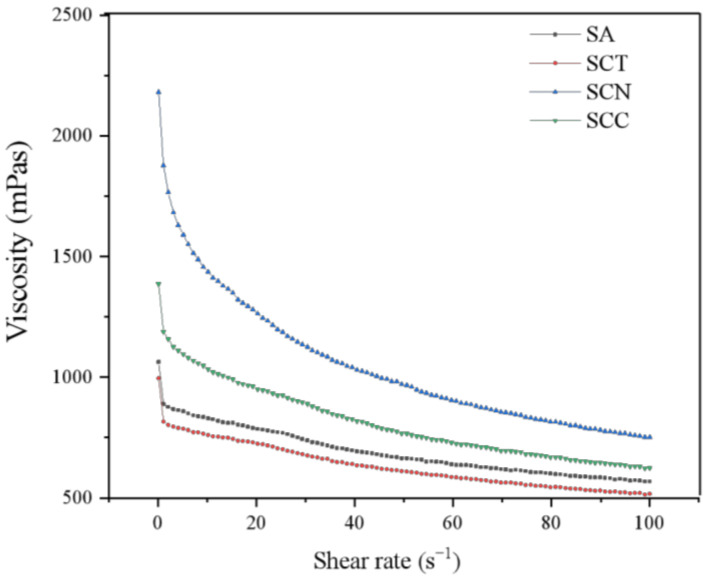
Flow curves of SA (sodium alginate), SCT (SA/CCT), SCN (SA/CN), and SCC (SA/CN/CCT) solutions.

The rheological characteristics of wall material influence physical properties of microcapsules. Therefore, we investigated the rheological properties of different wall materials (Figure 3). All four pastes were categorized as non-Newtonian fluids with shear thinning specific to pseudoplastic fluids. With the increase in shear rate, the viscosity of SCT, SCN, and SCC showed a rapid decreasing trend compared with SA solution. The addition of CN significantly increased the viscosity, while the addition of CCT performed inversely. The possible reason might be that CN have large aspect ratio and the ability to form interpenetrating network structures by hydrogen bonding, which give CN both the rigidity of ordered (crystalline) regions and the flexibility of disordered (amorphous) regions; these CN network structures can unravel and align parallel to the flow direction when the CN is in suspension, which can be used to explain the high degree of shear thinning exhibited by the SCN solution in this study, a similar phenomenon is observed in other solutions [29]. As the viscosity reflects the molecular movement of a viscoelastic body, and different viscosities indicate differences in the molecular structure, long and heavily twisted molecular chains result in high flow resistance and viscosity [30]. Polymer solutions of lower viscosity easily form small beads during the gelation process, whereas higher viscosity solutions tend to form larger ones. Therefore, particle size bound up with wall material pastes viscosity, and this result finds support in beads sizes.

#### 3.2.3. Morphology of Beads

**Figure 4:**
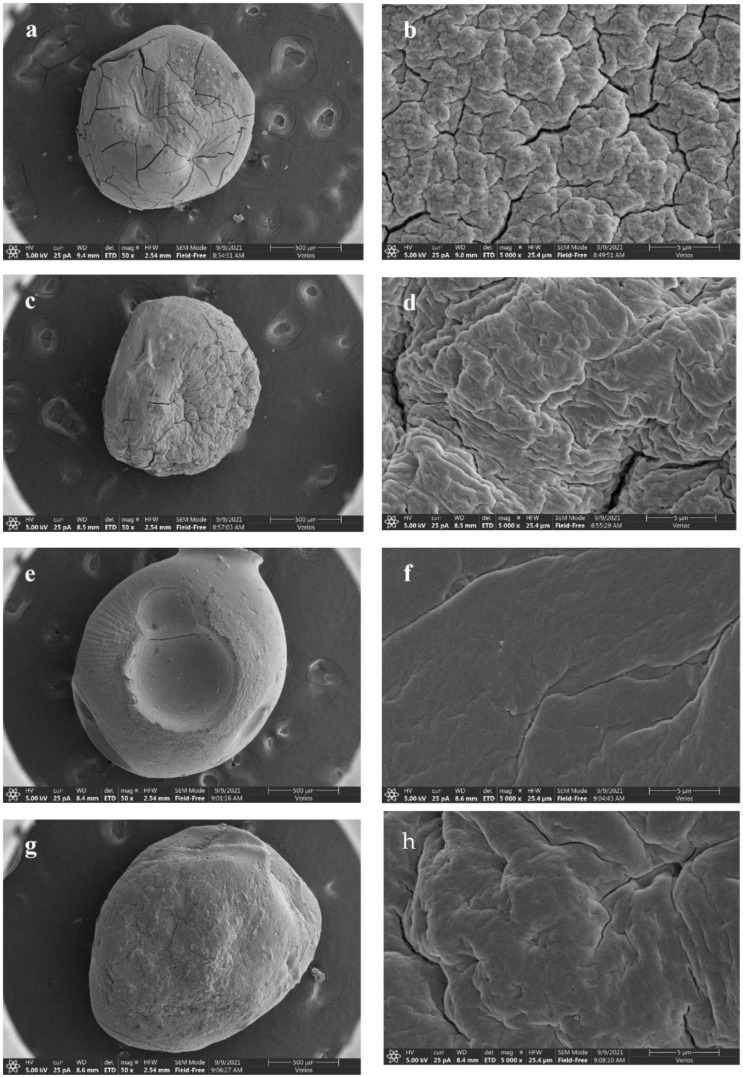
Scanning electron micrograph and surface morphology of (**a**,**b**) SA bead, (**c**,**d**) SCN bead, (**e**,**f**) SCT bead, and (**g**,**h**) SCC bead.

The morphology of lyophilized beads was observed by SEM (Figure 4). All kinds of dry beads have diameters in the range of 1300–1800 μm. During the dehydration, the beads formed irregular, uniform spheres with no aggregation, as the wall material shrank inward and collapsed partially. The surface texture of SA bead was rough, with some cracks and wrinkles (Figure 4a,b). The addition of CN narrowed the cracks and made the surface dense (Figure 4c,d). Images of the SCN and SCC beads showed that the beads did not shrink or swell with core-removal, likely due to the mechanical stability provided by the CN network [31]. The surface became smoother and denser by the addition of CCT (Figure 4a,e,g) with fewer cracks. Due to the polyhydroxy hydrophilic structure of CN, the pores and cracks on the bead surface did not improve after the beads had undergone the vacuum freeze-drying process; therefore, the SCT bead without CN showed a better performance in terms of surface density, and the same results were reported by Zhang et al. [32], who found the gel spheres would wrinkle with a higher CNF content. The sizes of beads were in the range of 1.5 ± 0.2 mm. The SCT bead was the largest, while the SCC bead and SCN bead were smaller, which indicated that the introduction of CN and CCT could enlarge the size of beads. This result was consistent with previous findings Shi et al. [33] and Li et al. [14]. Under 5000× magnification, it is clear that the addition of CCT formed a complete interpenetrating network, smoothing the originally SA surface (Figure 4b,f,h). These changes might result from the interactions between CCT chains and SA chains as well as the interactions between CCT chains and CN chains from interpenetrating and physical interweaving [22]. According to Shi et al. [33], the SA bead changed from a cracked, homogeneous structure to a core-layer and density model after blending with CCT. This effect was believed to play an important role in its release behavior.

#### 3.2.4. FT-IR

**Figure 5:**
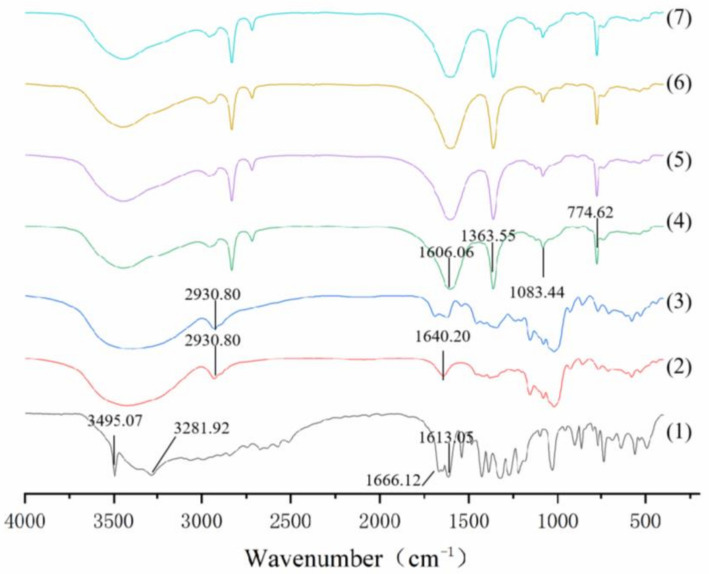
FT-IR spectra of (1) GA, (2) PS, (3) PG, (4) SA/PG beads, (5) SCN/PG beads, (6) SCT/PG beads, and (7) SCC/PG beads.

The FT-IR spectra of PS, GA, PG, as well as four types of beads are presented in Figure 5. The characteristic peaks of PS showed no obvious change before and after the adsorption of GA, which was because the adsorption process did not result in any molecular structure changes of the starch [24]. Therefore, there were no new chemical bonds formed between GA and PS. The results suggested that GA was mainly adsorbed through nonbonded interactions, such as the hydrogen bond’s interaction with PS [34]. In addition, the FT-IR spectra of the four beads showed strong and broad absorption bands in the range of 3100–3500 cm^−1^, indicating the large quantities of associated hydroxyl groups in these molecules [35]. The FT-IR results indicated the molecular compatibility among SA, CN, and CCT.

#### 3.2.5. Thermostability

**Figure 6:**
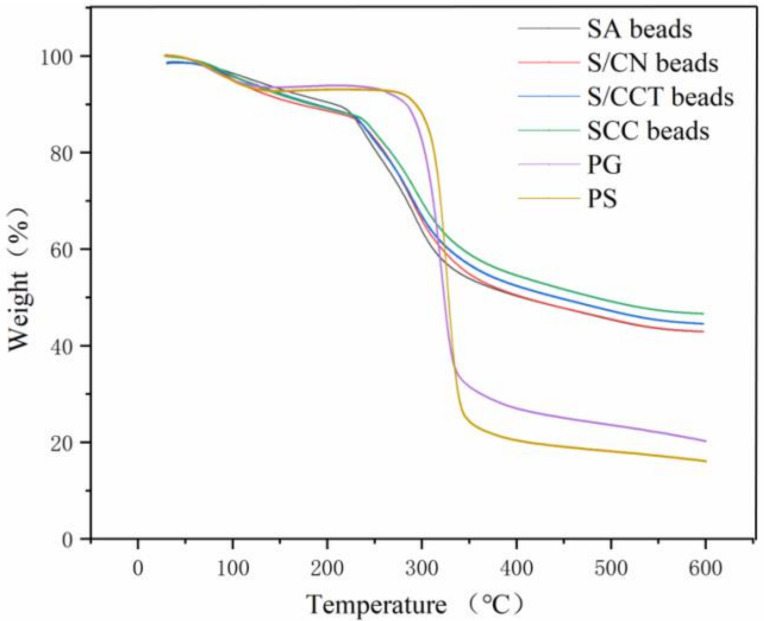
TG curves of PS, PG, SA beads, SCN beads, SCT beads, and SCC beads.

The thermogravimetric (TG) curves for the PS, PG powders, and four types of beads are displayed in Figure 6. Both PS and PG powders began to lose weight at about 100 °C, which was ascribed to the loss of bound water [36]. The cracking temperature of PG was approximately 300 °C, and the cracking process was not completed step by step, which might indicate that GA was adsorbed in the internal pores of PS. Additionally, the pyrolysis rate and the pyrolysis temperature of the PS obtained after the addition of GA was lower, which proved that GA entered the porous structure and interacted with starch through intermolecular forces. The weight losses of the four types of beads were observed in three degradation stages: (i) the mass of SA beads, S/CN beads, S/CCT beads, and SCC beads decreased from 50 °C to 230 °C by 13.3%, 13.2%, 12.8%, and 12.4%, respectively, as a result of water evaporation; (ii) from 230 °C to 330 °C, mass decreased by 43.7%, 41.9%, 40.2%, and 38.0%, respectively; and (iii) from 330 °C to 600 °C, mass decreased by 57.2%, 57.1%, 55.5%, and 53.3%, respectively. The last two stages corresponded to the release of additional water bound through polar interactions with the carboxylate, amine, and sulfate groups of SA, CNF, and CCT, respectively, as well as decomposition of the cyclic products and subsequent release of CO_2_ molecules from polysaccharides [22].

The formation of PIPNs improved the thermal stability of the beads relative to the original materials because of the cross-linking between SA and Ca^2+^ and the favorable electrostatic interactions between the opposite charges of the individual biopolymers. Further evidence was that the temperature required for half weight loss increased from approximately 400 °C for SA beads to 442 °C for the SCT beads and to 480 °C for the SCC beads. These differences between the thermograms of beads and pure native polymers confirm the presence of ionic interactions that could bring about the formation of new physical structures (PIPNs) with different thermal characteristics.

### 3.3. Texture Analysis

The definition and numerical calculation method of each index in Table 2 refers to the specification of the instrument. The value where the maximum deformation occurred during the first compression is the hardness of the beads, which reflects the gel strength of the beads and also represents the number and compactness of the gel network structure of wall materials. The product of hardness and cohesion, the value of which can be expressed as stickiness, is the energy required to chew a semi-solid food to a swallowing state. Resilience refers to the ratio of the height or volume of the deformable sample to the pre-deformation condition after the deformation force is removed by compression [37,38].

Table 3 shows that CN played an important role in all textural parameters studied. All the samples showed significant difference (*p*  <  0.05) in hardness. Additionally, these parameters, mainly hardness, increased with the addition of CN. Since CN can provide hydroxyl groups, which contribute to the stability of calcium alginate cross-linking, the hydroxyl groups in CN can form hydrogen bonds with water molecules and then fix free water, resulting in a polymer environment for gelation, which affects the textural properties of the bead [39].

It has been reported that the gel strength of hydrophilic colloids such as pectin [31] and protein [17] can be enhanced by the non-covalent binding force spring from the polyhydroxy structure of CN [12]. With better mechanical properties, the beads are more likely to stay intact after passing through the GI tract.

### 3.4. Swelling Rate

**Figure 7:**
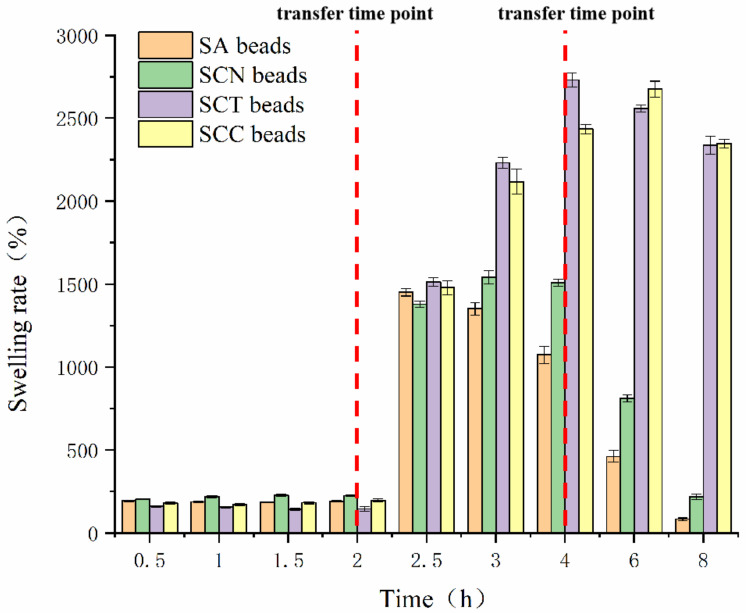
The effect of pH on the swelling rate of SA, SCN, SCT, and SCC beads.

Generally, the swelling behavior of the wall material of the beads is regarded as a manifestation of the pH sensitivity of the beads [40]. Figure 7 shows the swelling rates of the beads prepared from different wall materials in different pH buffers. After being soaked at buffer (pH 1.2) for 0.5 h, the wall materials showed different degrees of swelling behavior. The maximum expansion rate is 203.80%, and the expansion rate of the remaining beads is also greater than 150%. After being immersed at buffer (pH 1.2) for 2 h, the swelling ratio of the beads did not increase significantly compared to 0.5 h. The swelling behaviors might because strong hydrogen bonds form between the -COOH and -OH groups of CCT and CN and the -OH groups of SA and other -OH groups in the solution when the samples were immersed in the acidic medium [15]. The swelling ratio of the beads greatly increased when transferred to buffer (pH 6.8). After 0.5 h, the swelling ratio of SCN beads was the lowest (1378.60 ± 19.08%), whereas the highest ratio was the SCT beads (1512.30 ± 26.94%). With the extension of time, the expansion rate of all beads showed an upward trend. After being soaked for 2.5 h, the swelling rate of SA beads reached its highest point; after being soaked for 3, 4, and 6 h, the SCN, SCT, and SCC beads reached their highest points, respectively.

After reaching the maximum swelling, the wall materials began to fall apart. The SA beads cracked in the fastest and most thorough manner, with losing nearly 20% of their mass after 6 h. Similar swelling behavior of SA was reported by George et al. [41]. Likewise, the other three kinds of beads showed similar behavior but with delayed initial disintegration times and reduced degrees of fragmentation. The dramatic swelling of SCT beads and SCC beads could result from greater repulsion among CCT chains with -COO^−^ groups, as the -COOH groups are more deprotonated under alkaline environment. Meanwhile, the -COOH groups of alginate could also be ionized, providing a more negatively charged surface to the alginate molecule [15]. Therefore, the results implied that the wall material consisting of sodium alginate/carboxymethyl chitosan/cellulose nanofiber (S/C/C) was pH-sensitive and that the beads made by S/C/C could be used for delivering drugs or food ingredients that are unstable in the GI tract.

### 3.5. Release Analysis

**Figure 8:**
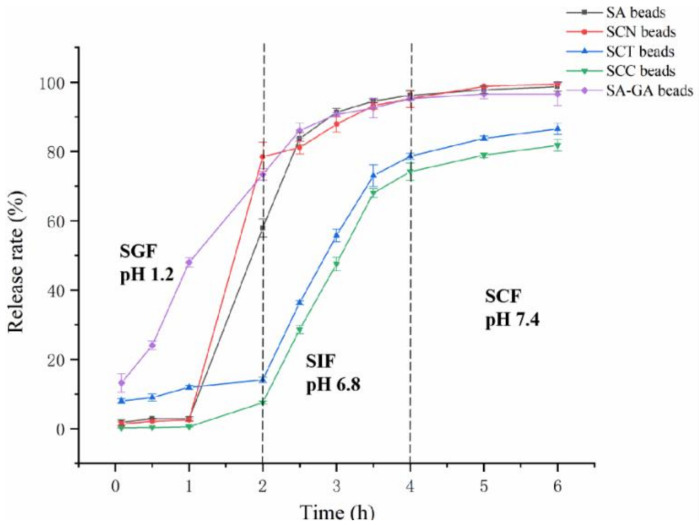
Cumulative release profiles of GA from the beads in SGF (pH 1.2), SIF (pH 6.8), and SCF (pH 7.4). SA, SCN, SCT, and SCC beads were loaded with PG. SA-GA beads were loaded with GA.

The cumulative release profiles of GA from five kinds of beads were investigated, which were shown in Figure 8. The release rates of GA from SCC and SCN beads in SGF were 7.84% and 78.49%, respectively.

This fits well with our previous report [18] and can be explained by the attachment of some GA to the surface of the beads, which are covered by an extremely thin layer of polymer. Another similar release behavior was observed according to Lopes et al. [42] study: nisin was rapidly released from phosphatidylcholine, phosphatidylcholine-pectin, and phosphatidylcholine-polygalacturonic acid nanoliposomes during the initial phase of the in vitro digestion simulation assay. Meanwhile, it was clear that the release rates of GA from SCT and SCC beads in SGF were much lower than SA and SCN beads. A possible reason might be the stable structure of core and the dense layer prevent the beads from disintegration. The release of GA was further observed for all formulations in SGF and eventually plateaued in SCF. Approximately 20% of GA was retained in SCC beads, whereas only less than 5% were retained in the SA, SCN, and SA-GA beads. The results support the notion that CCT strengthens the gel structure and helps realize sustained release of GA. Additionally, the release process may be related to matrix swelling, as we discussed in Section 3.4, and the swelling behavior of the wall material is regarded as a manifestation of the pH sensitivity, which could speed up/slow down the GA release process [40].

### 3.6. Antioxidant Activity of PG and Beads

The encapsulation efficiency and PI of all types of beads are shown in Table 4. After dehydration and suspension of the beads in water for 4 h, GA was released in suspension. Given that the wall material underwent structural degradation after suspension in water, the value of PI in suspension reflected the protective efficiency of the wall material to the contents. Hence, by measuring the PI of suspension, the protective activities of different beads to GA could be verified. There were significant differences in the antioxidant activities of the beads. The SCC beads showed the lowest antioxidant capacity but the highest encapsulation efficiency, which demonstrated the addition of CCT and CN with SA, as the wall material did play a role in the protectiveness of GA in order to maintain the bioavailability during the delivery process.

Da Rosa et al. [43] studied the encapsulation of GA by chitosan, β-cyclodextrin, and xanthan gum, and the encapsulated GA showed no loss of antioxidant capacity and different characteristics from the pure GA, confirming the techniques used. Sun et al. [44] studied the microencapsulation and antimicrobial activity of carvacrol in a pectin-alginate matrix, and after destroying the pectin-alginate matrix, the free radical inhibition percentages for encapsulated phenolic extract still reached 89.96%, which was similar to the results in this study.

## 4. Conclusions

In the study, gallic acid was successfully loaded into PS, and the SCC beads containing the GA-loaded PS successfully traversed the simulated stomach, indicating that the delivery system constructed in this study is able to protect GA from extreme pH environments and that the majority of the loaded GA could reach the small intestine. This study demonstrates the effectiveness of this biocompatible drug delivery system. The dosage of PG powder added to the coating solution could be increased, and the beads can be freeze-dried, allowing further applications for the addition of bioactive molecules in food. This study provides basic theoretical information for the design of small-intestine-targeted delivery systems using PS and SCC as coating layers. In addition, this system can be used to deliver other bioactive molecules with poor oxidative stability in acidic environments and high solubility in aqueous solutions.

## Figures and Tables

**Table 1:** Coded values and corresponding actual values of the optimization parameters used in response surface analysis.

Level	Factors			
	A-CaCl_2_ (%)	B-SA (%)	C-CN (%)	D-CCT (%)
−1	1.0	1.5	0.4	0.5
0	2.0	2.0	0.5	1.0
1	3.0	2.5	0.6	1.5

SA: sodium alginate, CN: cellulose nanofibers, CCT: carboxymethyl chitosan.

**Table 2:** Analysis of variance for encapsulation efficiency of beads.

Source	Sum of Square	df	Mean Square	F-Value	*p*-Value	Significance ^a^
Model	1381.7	22	62.8	9.05	0.0057	***
A-CaCl_2_	41.22	1	41.22	5.94	0.0507	*
B-SA	55.8	1	55.8	8.04	0.0297	**
C-CN	149.82	1	149.82	21.59	0.0035	***
D-CCT	62.25	1	62.25	8.97	0.0241	**
AB	77.35	1	77.35	11.15	0.0156	**
AC	10.63	1	10.63	1.53	0.2621	*
AD	102.62	1	102.62	14.79	0.0085	***
BC	2.81	1	2.81	0.4044	0.5483	*
BD	4.45	1	4.45	0.6417	0.4536	*
CD	4.49	1	4.49	0.6478	0.4516	*
A^2^	427.04	1	427.04	61.55	0.0002	***
B^2^	151.8	1	151.8	21.88	0.0034	***
C^2^	112.6	1	112.6	16.23	0.0069	***
D^2^	0.3073	1	0.3073	0.0443	0.8403	*
A^2^B	27.86	1	27.86	4.02	0.0919	*
A^2^C	54.81	1	54.81	7.9	0.0307	**
A^2^D	44.18	1	44.18	6.37	0.0451	**
AB^2^	0.3741	1	0.3741	0.0539	0.8241	*
AC^2^	0.4608	1	0.4608	0.0664	0.8052	*
B^2^C	47.09	1	47.09	6.79	0.0404	**
B^2^D	50.1	1	50.1	7.22	0.0362	**
BC^2^	11.45	1	11.45	1.65	0.2463	*
Residual	41.63	6	6.94			
Lack of Fit	16.79	2	8.4	1.35	0.3559	*
Pure Error	24.83	4	6.21			
Cor Total	1423.33	28				
R_2_			0.9708			
Adj R_2_			0.8635			
Pred R_2_			−0.7264			

Adequate precision = 11.3654, cv = 5.50%. ^a^ * No significant difference (*p* > 0.05), ** Significantly different (*p* < 0.05), *** Extremely significantly different (*p* < 0.01).

**Table 3:** Texture profile analysis of beads.

Samples	Hardness (g)	Adhesiveness (g·s)	Resilience	Cohesiveness	Springiness	Gumminess (N)
SA beads	84.28 ± 1.01 ^c^	−0.35 ± 0.02 ^a^	25.59 ± 1.23 ^a^	0.58 ± 0.03 ^a^	70.94 ± 0.30 ^a^	48.81 ± 3.17 ^c^
SCN beads	72.90 ± 5.29 ^c^	−0.40 ± 0.13 ^a^	25.32 ± 0.49 ^a^	0.58 ± 0.02 ^a^	74.16 ± 5.58 ^a^	41.97 ± 3.08 ^c^
SCT beads	102.32 ± 6.24 ^b^	−0.11 ± 0.36 ^a^	22.24 ± 1.21 ^b^	0.54 ± 0.01 ^a^	57.47 ± 4.85 ^b^	55.26 ± 3.33 ^b^
SCC beads	147.15 ± 5.47 ^a^	−0.63 ± 0.65 ^a^	25.01 ± 0.82 ^a^	0.59 ± 0.02 ^a^	62.49 ± 2.70 ^b^	86.11 ± 3.10 ^a^

Values in the same column with different letters are significantly different (*p* < 0.05).

**Table 4:** Encapsulation efficiency and antioxidant activity by DPPH (2,2-diphenyl-1-picrylhydrazyl) method.

	Encapsulation Efficiency (%)	Percentage of Inhibition (PI) (%)
GA		94.90 ± 0.86
PG		91.47 ± 0.70
SA beads	40.61 ± 0.68	87.16 ± 0.33
SCN beads	42.36 ± 0.73	82.86 ± 1.09
SCT beads	48.65 ± 0.64	72.48 ± 1.19
SCC beads	56.46 ± 0.48	61.00 ± 0.99

## Data Availability

The data presented in this study are available in this article.
